# Advancing Diabetic Retinopathy Diagnosis: Leveraging Optical Coherence Tomography Imaging with Convolutional Neural Networks

**DOI:** 10.22336/rjo.2023.53

**Published:** 2023

**Authors:** H Shafeeq Ahmed, Chinmayee J Thrishulamurthy

**Affiliations:** * Department of Ophthalmology, Bangalore Medical College and Research Institute, Karnataka, India

**Keywords:** diabetic retinopathy, automated screening, multimodal imaging integration, real-time diagnosis, point-of-care systems, Convolutional Neural Networks

## Abstract

Diabetic retinopathy (DR) is a vision-threatening complication of diabetes, necessitating early and accurate diagnosis. The combination of optical coherence tomography (OCT) imaging with convolutional neural networks (CNNs) has emerged as a promising approach for enhancing DR diagnosis. OCT provides detailed retinal morphology information, while CNNs analyze OCT images for automated detection and classification of DR. This paper reviews the current research on OCT imaging and CNNs for DR diagnosis, discussing their technical aspects and suitability. It explores CNN applications in detecting lesions, segmenting microaneurysms, and assessing disease severity, showing high sensitivity and accuracy. CNN models outperform traditional methods and rival expert ophthalmologists’ results. However, challenges such as dataset availability and model interpretability remain. Future directions include multimodal imaging integration and real-time, point-of-care CNN systems for DR screening. The integration of OCT imaging with CNNs has transformative potential in DR diagnosis, facilitating early intervention, personalized treatments, and improved patient outcomes.

**Abbreviations:** DR = Diabetic Retinopathy, OCT = Optical Coherence Tomography, CNN = Convolutional Neural Network, CMV = Cytomegalovirus, PDR = Proliferative Diabetic Retinopathy, AMD = Age-Related Macular Degeneration, VEGF = vascular endothelial growth factor, RAP = Retinal Angiomatous Proliferation, OCTA = OCT Angiography, AI = Artificial Intelligence

## Introduction

Diabetic retinopathy (DR) is a prevalent and potentially sight-threatening complication of diabetes. Early and accurate diagnosis is crucial for timely intervention and effective management of this condition [**1**]. Approximately one-third of the estimated 285 million people with diabetes mellitus worldwide have signs of DR), and within this group, a further one-third of DR cases are classified as vision-threatening DR, including diabetic macular edema [**2**]. Optical coherence tomography (OCT) imaging has emerged as a powerful diagnostic tool, offering high-resolution and non-invasive visualization of retinal structures. On the other hand, convolutional neural networks (CNNs) have shown remarkable success in image analysis and pattern recognition tasks [**3**].

The integration of OCT imaging with CNNs holds great promise in enhancing diagnostic accuracy, efficiency, and timeliness. OCT provides detailed and quantitative information about retinal morphology, enabling the identification of early signs of DR and precise characterization of disease progression [**4**-**6**]. With their ability to learn complex features and patterns from large datasets, CNNs can analyze OCT images and assist in automated detection, classification, and risk stratification of DR [**7**].

This paper aims to review the current state of research and development in using OCT imaging with CNNs for DR diagnosis. It discusses the technical aspects of OCT imaging, the principles of CNNs, and their suitability for analyzing OCT images [**3**,**5**]. Furthermore, it explores the various applications of CNNs in DR diagnosis, including lesion detection, microaneurysm segmentation, and disease severity assessment. 

By leveraging the complementary strengths of OCT imaging and CNNs, we can envision a future in which DR diagnosis is more accurate, efficient, and accessible. This novel approach has the potential to revolutionize clinical practice, enabling early intervention and personalized treatment strategies. Ultimately, it aims to improve patient outcomes and reduce the burden of DR on individuals and healthcare systems.

## OCT and DR

**Fig. 1:**
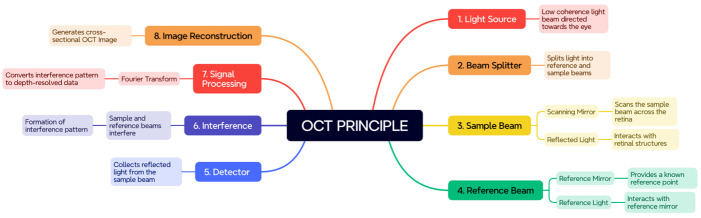
OCT Imaging Principle

OCT has revolutionized the field of ophthalmic imaging by providing non-invasive, high-resolution cross-sectional images of the retina [**8**]. It utilizes the principles of interferometry to capture detailed structural information of retinal tissue. OCT imaging techniques involve directing a low-coherence light source toward the eye, which is then split into a reference beam and a sample beam [**9**,**10**]. The reflected light from the retina is collected, interfered with the reference beam, and analyzed to reconstruct the retinal image. **Fig. 1** illustrates the fundamental principles of OCT mechanics, which support its application in ophthalmology.

In DR diagnosis, OCT imaging plays a crucial role in assessing retinal morphological changes associated with the disease [**11**]. By employing specific acquisition protocols, such as the macular cube or line scans, OCT can generate detailed images of the macula and optic nerve head region, enabling the identification of abnormalities and quantitative assessment of retinal thickness [**12**,**13**].

**Table 1:** Advantages, disadvantages, and alternative options for OCT in diabetic retinopathy diagnosis

Advantages	Details	Disadvantages	Details	Alternative Options
High-Resolution Imaging	OCT provides detailed cross-sectional images of retinal structures, allowing for precise visualization of abnormalities	Cost of Equipment	OCT machines can be expensive to purchase, and regular maintenance is required, which can be costly	Alternative imaging technologies: Fundus photography, Ultrasonography, or smartphone-based retinal imaging devices
Non-Invasive Technique	OCT is non-invasive, involving no contact with the eye, reducing patient discomfort and the risk of infection	Limited Access in Some Settings	Smaller healthcare facilities or underserved areas may lack access to OCT technology	Mobile or telemedicine units equipped with alternative imaging tools, or scheduled access to nearby healthcare facilities with OCT machines
Quantitative Assessment	OCT allows for quantitative measurement of retinal thickness, which aids in tracking disease progression	Skilled Technician Requirement	Skilled technicians are needed to operate OCT equipment, which may not be available everywhere	Implement training programs for technicians and healthcare professionals or explore the use of automated systems with user-friendly interfaces
Precise Monitoring of Progression	OCT is valuable for monitoring subtle changes over time, providing an early indication of disease progression	Patient Cooperation	Patients need to cooperate by holding still, which can be challenging for some, particularly young or elderly patients	Implement devices with fixation aids, or utilize sedation or anesthesia for patients with limited cooperation
Early Detection of Subtle Changes	OCT enables the early detection of retinal changes, facilitating early intervention and better patient outcomes	Possible Artifacts	Eye movement can lead to artifacts or distortions in the images, affecting diagnostic accuracy	Develop eye-tracking systems to reduce artifacts or combine OCT with other imaging modalities to cross-verify findings
Individualized Treatment Planning	Detailed imaging data helps ophthalmologists create personalized treatment plans tailored to each patient’s needs	Limitations in Imaging	OCT may not effectively capture deeper retinal layers, potentially missing important pathology	Use complementary imaging techniques, like fundus fluorescein angiography or Magnetic Resonance Imaging, to visualize deeper retinal layers
Integration with Advanced Image Analysis Techniques	Integration with techniques such as CNNs enhances diagnostic accuracy by automating the analysis of OCT images	Interpretation Expertise	Accurate interpretation of OCT images often requires specialized expertise in retinal pathology	Utilize computer-aided diagnosis (CAD) systems or telemedicine for remote consultation with experts in retinal pathology

Retinal layer segmentation is an essential step in OCT image analysis for DR diagnosis [**13**]. Advanced algorithms are used to accurately segment individual retinal layers, including the retinal nerve fiber layer, ganglion cell layer, inner plexiform layer, inner nuclear layer, outer plexiform layer, and photoreceptor layer [**14**-**16**]. The thickness and integrity of these layers provide valuable information about the structural changes occurring in DR.

Moreover, OCT-based biomarkers have been identified to aid in the detection and monitoring of DR. Biomarkers such as central macular thickness, macular volume, foveal thickness, and the presence of intraretinal cysts, subretinal fluid, or drusen-like deposits can serve as indicators of disease severity and progression [**17**,**18**]. Quantitative measurements derived from OCT images, such as the thickness of the ganglion cell-inner plexiform layer complex or the presence of hyperreflective foci, have shown promise as prognostic markers for DR. **Table 2** presents the hallmark OCT features observed in DR, along with their potential differentials. Additionally, it highlights the rarer findings and provides alternative evaluation methods to confirm or differentiate these findings from DR.

**Table 2:** OCT findings in diabetic retinopathy and their possible differential diagnoses

Hallmark Features	Possible Differentials	Rarer Findings	Alternative Evaluation
Macular Edema	- Retinal vein occlusion - Age-related macular degeneration (AMD) - Retinitis pigmentosa	- Cystoid macular edema	- Fluorescein angiography for confirmation
Hard Exudates	- Retinal vein occlusion - AMD - Retinitis pigmentosa - Cytomegalovirus (CMV) retinitis	- Drusen	- Fundus photography for confirmation
Intraretinal Hemorrhages	- Retinal vein occlusion - Hypertensive retinopathy - Coagulopathy	- Flame-shaped hemorrhages	- Clinical history and systemic evaluation
Cotton Wool Spots	- Hypertensive retinopathy - Retinal vein occlusion - HIV-associated retinopathy	- Roth spots	- Clinical history and systemic evaluation
Epiretinal Membrane	- Idiopathic epiretinal membrane - Macular hole - Vitreomacular traction syndrome	- Contractile epiretinal membrane	- Surgical evaluation for membrane removal
Neovascularization	- Proliferative diabetic retinopathy (PDR) - AMD - Retinal vein occlusion - Retinal artery macroaneurysm	- Retinal angiomatous proliferation (RAP)	- Intravitreal anti-vascular endothelial growth factor (VEGF) agents
Atrophy of the Outer Retina	- Geographic atrophy in AMD - Retinitis pigmentosa - Cone-rod dystrophy	- Parafoveal hyperpigmentation	- Electroretinography for retinal function assessment
Vitreous Hemorrhage	- PDR - Retinal vein occlusion - Terson syndrome (subarachnoid hemorrhage)	- Terson syndrome	- Detailed medical history and intracranial imaging

The non-invasive nature, high-resolution imaging, and quantitative capabilities of OCT make it an invaluable tool for DR diagnosis. It allows for early detection of subtle retinal changes, precise monitoring of disease progression, and individualized treatment planning [**19**]. The integration of OCT imaging with advanced image analysis techniques, such as CNNs, holds significant potential to further enhance the accuracy and efficiency of DR diagnosis, ultimately leading to improved patient care and outcomes [**20**].

## CNNs and automated image analysis

CNNs have emerged as a state-of-the-art deep learning technique for automated image analysis. Unlike traditional machine learning algorithms, CNNs can automatically learn and extract complex features directly from raw image data [**21**,**22**]. This makes them highly suitable for processing and analyzing medical images, including OCT scans in DR diagnosis. 

The architecture of CNNs is designed to mimic the visual processing mechanism of the human brain. It consists of multiple layers, including convolutional layers, pooling layers, and fully connected layers [**23**]. Convolutional layers perform feature extraction by applying filters to capture local patterns and features in the input images. Pooling layers reduce the spatial dimensions of the extracted features, allowing for hierarchical feature representation. Fully connected layers then process these features to make predictions based on learned patterns [**20**]. 

Training CNNs involves feeding a large dataset of labeled images into the network and adjusting the weights and biases through a process called backpropagation. This allows the network to learn the optimal set of parameters that can accurately classify or segment images [**24**]. The training process often requires substantial computational resources and may involve techniques such as data augmentation, regularization, and optimization algorithms to improve performance and prevent overfitting [**25**,**26**]. 

**Table 3:** Components and configuration of CNNs for diabetic retinopathy diagnosis

Layer/Component	Description/Alternatives	Use Cases/Considerations
Input Layer	Input image size (e.g., 128x128)	Initial image dimensions (pixel size) for model input. It is typically a square image, e.g., 128x128 pixels, but can vary based on data and computation resources.
Convolutional Layers	Number of layers (3-5), filter size, padding, and strides	These layers extract patterns and features from images. The number of layers, the size of filters (small grids that scan the image), padding (adding zeros to the image border), and strides (step size for the filter) influence feature extraction.
Pooling Layers	Type (max-pooling, avg-pooling), number of layers, pooling window size	Pooling layers down-sample and reduce spatial dimensions. Max-pooling selects the maximum value within a window, while avg-pooling computes the average. The number of layers and window size affect spatial resolution.
Batch Normalization	After convolutional and fully connected layers	Normalization technique to improve training speed and model generalization. Applied after convolutional and fully connected layers to normalize layer inputs.
Dropout Layers	After fully connected layers, the dropout rate	Dropout layers randomly drop a fraction of neurons during training to prevent overfitting. The dropout rate controls the fraction of neurons to be dropped.
Fully Connected Layers	Number of layers (1-3), number of neurons per layer (e.g., 128, 256, 512)	These layers perform the final classification. The number of layers and neurons per layer determines the network’s capacity.
Output Layer	Single neuron, sigmoid activation	The output layer has a single neuron and a sigmoid activation function for binary classification, in which the output represents the likelihood of having diabetic retinopathy.
Loss Function	Binary Cross-Entropy	A loss function measures the model’s prediction error. Binary Cross-Entropy is commonly used for binary classification tasks. It quantifies the difference between predicted and actual classes.
Optimizer	Adam, RMSprop, SGD	Optimizers adjust model parameters during training. Adam, RMSprop, and SGD are optimization algorithms. They control how the model learns from data.
Data Augmentation	Techniques like rotation, flipping, cropping	Data augmentation artificially expands the training dataset by applying transformations like rotation and flipping to images. This enhances model robustness and generalization.
Transfer Learning	Pre-trained models (e.g., VGG, ResNet)	Transfer learning leverages pre-trained models (trained on large datasets) and fine-tunes them for specific tasks. It accelerates training and improves performance.
Hyperparameter Tuning	Learning rate, batch size, dropout rate	Hyperparameters are settings for training. The learning rate controls the step size during optimization. The batch size determines the number of samples processed at once. Dropout rate manages dropout layer behavior.
Model Evaluation	Metrics like accuracy, precision, recall, F1-score, AUC-ROC	These evaluation metrics assess model performance. Accuracy is the overall correctness. Precision measures true positives among predicted positives. Recall (sensitivity) measures true positives among actual positives. F1-score combines precision and recall. AUC-ROC is the area under the receiver operating characteristic curve.
Ensemble Methods	Combining predictions from multiple models	Ensemble methods involve aggregating predictions from multiple models to improve robustness and accuracy.

In the context of DR diagnosis using OCT images, CNNs need to be adapted and optimized to effectively analyze the unique characteristics of OCT scans [**10**]. This includes pre-processing techniques to enhance image quality, such as denoising and contrast enhancement. Additionally, specific modifications to the network architecture and training strategies may be required to handle the high-resolution volumetric data obtained from OCT scans [**27**].

## Integrating OCT & CNNs for DR

Gulshan et al. demonstrated the potential of a deep CNN algorithm in detecting referable DR and diabetic macular edema [**28**]. The algorithm achieved high sensitivity and specificity, comparable to expert ophthalmologists, indicating its effectiveness as a screening tool.

Li et al. focused on the detection of diabetic macular edema using OCT images [**29**]. Their CNN-based algorithm outperformed traditional methods, achieving a high accuracy of 94.5%. This highlights the superiority of CNNs in improving diagnostic accuracy for specific DR lesions. 

Yashasvini et al. developed and compared different CNN architectures for automated DR detection and classification [**30**]. The CNN, hybrid CNN with ResNet, and hybrid CNN with DenseNet achieved accuracies of 96.22%, 93.18%, and 75.61%, respectively. This indicates that the choice of CNN architecture can affect the performance of the algorithm. 

Reguant et al. (2019) focused on the clinical relevance of the image features learned by CNNs in DR detection [**6**]. They found that CNN-based methods achieved high accuracy, sensitivity, and specificity for grading the disease level of DR. Additionally, the CNN visualization strategy provided insights into the image features important for decision-making. 

**Table 4** offers an overview and comparison of various hybrid CNN models commonly employed in medical imaging tasks within ophthalmology, addressing specific needs, key features, and situations in which each model excels. These models encompass a range of architectural innovations and are tailored to different scenarios within ophthalmic image analysis, from general tasks to specialized applications. Each model’s key component is highlighted, followed by a brief description of its features and potential use cases.

**Table 4:** Comparison of Hybrid CNN Models for Medical Imaging

Model	Key Component (s)	Suitable for...	Key Features	When to Use	When Not to Use
ResNet	Residual Blocks	Various medical imaging tasks	Use shortcuts to prevent issues with deep networks	For general medical imaging tasks, such as...	Not suitable for real-time applications due to depth
DenseNet	Dense Blocks	Early disease diagnosis	Connect layers for feature reuse.	Medical diagnosis, ophthalmic image analysis...	May require substantial memory for large datasets
Inception V3	Inception Blocks	Fine-grained classification	Utilize various filter sizes for deep networks	Fine-grained image recognition, pathology analysis...	May be complex for simpler image classification
VGG16	Convolutional Layers	General image recognition	Employ simple 3x3 filters with deep architecture	General image classification, glaucoma detection...	May not suit real-time applications due to the complexity
MobileNet	Depthwise Separable Convolution	Mobile devices and edge computing	Use lightweight depthwise separable convolutions	Mobile and edge applications, real-time analysis...	Not ideal for complex image analysis tasks
Xception	Separable Convolution	Fine-grained classification	Substitute standard convolutions for efficiency	Fine-grained image recognition, pathology analysis...	May be too complex for simpler image classification.
SqueezeNet	Fire Modules	Resource-constrained devices	Compact architecture with 1x1 convolutions	Mobile devices, resource-constrained environments...	May not handle high-resolution images efficiently
NASNet	Neural Architecture Search	Various medical tasks	Designed via neural architecture search for efficiency	Medical image analysis, glaucoma detection...	May not be suitable for resource-constrained devices
Inception-ResNet-V2	Inception Blocks, Residual	Fine-grained classification	Combines Inception and ResNet for deep efficiency	Fine-grained image recognition, pathology analysis...	May be overkill for simpler image recognition tasks
ResNeXt	Residual Blocks, Split-Transform	Image classification, object detection	Employs split-transform strategy for scaling up	General image classification, glaucoma detection...	Requires a substantial dataset for maximizing potential
ShuffleNet	Pointwise Group Convolutions	Low-latency and high-performance	It uses pointwise group convolutions for efficiency	Real-time applications, resource-constrained devices...	May not be ideal for complex medical imaging tasks
SENet	Squeeze-and-Excitation Blocks	Fine-grained classification	Enhances feature recalibration with SE blocks	Fine-grained image recognition, pathology analysis...	May be complex for simpler image classification tasks

Overall, these studies collectively demonstrate the potential of integrating OCT and CNNs for DR diagnosis. The use of CNN algorithms trained on large datasets of retinal images, including OCT scans, can achieve high accuracy, sensitivity, and specificity in detecting DR and its associated lesions. This technology can improve screening efficiency, enable early intervention, and enhance patient outcomes in DR management. However, further research and validation on larger and more diverse datasets are needed to establish the generalizability and clinical applicability of these findings.

## Performance utilization and clinical relevance

Several studies employed various imaging modalities, such as OCT, fundus photography, and OCT angiography (OCTA), to evaluate the performance of CNN models in real-world settings. 

**Table 5:** Comparison of fundus imaging, OCT, and OCT angiography in diabetic retinopathy

Characteristic	Fundus Imaging	Optical Coherence Tomography (OCT)	OCT Angiography
Imaging Technique	2D retinal photography	Cross-sectional imaging	Vascular and microvascular imaging
Information Provided	Anatomical retinal structure	Detailed retinal layers	Vascular and blood flow data
Primary Use	Assessment of retinal health	Diagnosing and monitoring changes	Detecting vascular abnormalities
Key Advantages	Non-invasive, quick	High-resolution structural images	Visualization of blood flow
Common Diabetic Retinopathy Applications	Monitoring retinal changes	Quantifying retinal thickness	Assessing neovascularization
Limitations	Limited depth information	Limited to structural data	It does not provide anatomical data
Use in Diabetic Retinopathy	Detecting retinal changes, assessing complications	Evaluating macular edema, assessing retinal layers	Detecting microaneurysms, assessing neovascularization

Ryu et al. developed an automated DR staging system using OCTA images and a deep CNN [**26**]. Their results demonstrated that the CNN algorithm outperformed traditional machine learning methods and human experts in accurately classifying DR stages. The CNN achieved high accuracy, sensitivity, specificity, F1 score, and weighted kappa score for the six-level staging task. These findings indicate that deep CNNs have the potential to accurately stage DR based on OCTA images, eliminating the need for manual grading by experts. 

Shaban et al. proposed a deep CNN model for objectively diagnosing and grading DR based on fundus images [**31**]. Their CNN model demonstrated high accuracy, sensitivity, specificity, and weighted kappa score, suggesting its potential as a diagnostic tool for DR. The proposed approach eliminates the need for a retina specialist, expanding access to retinal care and enabling early diagnosis and objective tracking of disease progression. 

The CNN models demonstrated significant advantages in terms of accuracy and performance compared to traditional methods and human experts [**26**-**31**]. They achieved high sensitivity, specificity, and accuracy in detecting DR and its associated lesions, such as macular edema and microaneurysms, using various imaging modalities like OCT, OCTA, and fundus photography. These findings indicate the potential of CNNs to serve as reliable and efficient tools for DR screening, enabling early detection and accurate classification of different disease stages. Additionally, CNNs offer the advantage of eliminating the need for manual grading by experts, expanding access to retinal care, and optimizing medical therapy to minimize vision loss. However, it is important to acknowledge the limitations of these studies. The retrospective nature of some studies and the variations in dataset sizes and characteristics may introduce biases and affect generalizability. The performance of CNNs may vary depending on the specific imaging modality, image quality, and dataset used. Further validation studies and large-scale clinical trials are needed to assess the real-world clinical relevance and impact of these CNN models on patient outcomes. Moreover, the interpretability of CNNs remains a challenge, as the black-box nature of these models limits the understanding of the underlying features driving their predictions.

## Challenges and future directions

While the studies discussed have shown promising results in utilizing CNNs for DR diagnosis, several challenges and limitations need to be addressed for further advancements in the field. One major challenge is the availability of larger and more diverse datasets [**20**]. The performance of CNN models heavily relies on the quality and representativeness of the training data. Obtaining a comprehensive dataset that covers a wide range of DR stages, variations in imaging modalities, and diverse patient populations is crucial to improving the generalizability and robustness of CNN models. Another challenge lies in the interpretability of CNN models. The black-box nature of deep learning algorithms makes it difficult to understand the underlying features and decision-making processes [**32**,**33**]. Addressing this challenge requires the development of explainable AI techniques that can provide insights into how CNN models arrive at their predictions. Techniques such as attention mechanisms and feature visualization can help in interpreting the learned features and increasing the transparency of the models. 

Furthermore, potential biases in training data need to be carefully addressed. Biases present in the data, such as underrepresented populations or imbalanced classes, can affect the performance and fairness of CNN models [**6**]. Efforts should be made to ensure that the training data is diverse and representative of the population to mitigate biases and improve the generalizability of the models. Emerging techniques offer potential solutions to overcome these challenges. Transfer learning, for instance, allows the knowledge gained from pretraining on large-scale datasets to be transferred and fine-tuned on specific DR datasets, thereby improving the performance of CNN models with limited data [**34**,**35**]. By leveraging the pre-learned features, transfer learning enables more efficient training and better generalization. In addition, the development of explainable AI techniques can enhance the interpretability of CNN models. Methods such as attention maps and saliency mapping can help identify the regions of the image that contribute most to the model’s decision, providing insights into the features and patterns that the model focuses on during classification [**36**-**38**].

Looking ahead, future research directions should explore the integration of multimodal imaging to improve DR diagnosis and monitoring [**39**,**40**]. Combining information from different imaging modalities, such as OCT, OCTA, and fundus photography, can provide a more comprehensive assessment of retinal changes and enhance the accuracy of DR classification. Furthermore, the development of real-time, point-of-care CNN systems based on OCT holds great potential. Such systems would enable immediate and efficient DR screening in primary care settings, enhancing access to timely diagnosis and intervention.

## Conclusion

In summary, the integration of OCT imaging with CNN has revolutionized the diagnosis of DR. The combination of OCT’s rich structural information and the deep learning capabilities of CNNs has resulted in accurate, efficient, and accessible detection of DR lesions. This advancement holds tremendous potential in improving patient outcomes by enabling early detection, timely intervention, and optimized medical therapy to minimize vision loss associated with DR. By leveraging the power of CNNs, these OCT-based algorithms have surpassed traditional methods and even demonstrated performance comparable to expert ophthalmologists in some cases. The high accuracy, sensitivity, and specificity achieved by these models highlight their clinical utility and impact. Moreover, the integration of CNNs with OCT has the advantage of being non-invasive, making it a valuable tool for routine screening and monitoring of DR. However, there are still challenges to address. One key area of improvement is the availability of larger and more diverse datasets, which would enhance the generalizability and robustness of CNN models. Additionally, interpretability remains a challenge with deep learning algorithms. Efforts should be made to develop explainable AI techniques that shed light on the decision-making processes of CNN models, increasing their transparency and trustworthiness. Future research should also focus on integrating OCT with other imaging modalities, such as OCT angiography and fundus photography, to provide a more comprehensive assessment of retinal changes. This multimodal approach could further enhance the accuracy and diagnostic capabilities of DR detection systems. Furthermore, the development of real-time, point-of-care CNN systems based on OCT holds great promise in expanding access to timely and efficient DR screening, particularly in primary care settings. In conclusion, the integration of OCT imaging with CNNs has transformed the landscape of DR diagnosis. The potential to improve patient outcomes, optimize medical therapy, and minimize vision loss associated with DR is substantial. Continued advancements in data availability, interpretability, and integration with other imaging modalities will pave the way for more personalized and effective management of this sight-threatening condition. The future of DR diagnosis is bright, offering hope for enhanced care and better quality of life for patients worldwide.

**Conflict of Interest Statement**

The authors declare that they have no conflict of interest.

**Acknowledgments**

None.

**Sources of Funding**

None.

**Disclosures**

None.
